# Diverse manifestations of the mid-Pleistocene climate transition

**DOI:** 10.1038/s41467-018-08257-9

**Published:** 2019-01-21

**Authors:** Youbin Sun, Qiuzhen Yin, Michel Crucifix, Steven C. Clemens, Pablo Araya-Melo, Weiguo Liu, Xiaoke Qiang, Qingsong Liu, Hui Zhao, Lianji Liang, Hongyun Chen, Ying Li, Li Zhang, Guocheng Dong, Ming Li, Weijian Zhou, Andre Berger, Zhisheng An

**Affiliations:** 1 State Key Laboratory of Loess and Quaternary Geology, Institute of Earth Environment, Chinese Academy of Sciences, 710061 Xian, China; 2 CAS Center for Excellence in Quaternary Science and Global Change, Chinese Academy of Sciences, 710061 Xian, China; 3 Georges Lemaître Center for Earth and Climate Research, Earth and Life Institute, Université Catholique de Louvain, 1348 Louvain-la-Neuve, Belgium; 4 Department of Earth, Environmental and Planetary Sciences, Brown University, Providence, 02912-1846 RI USA; 5 Centre for Marine Magnetism, Department of Ocean Science and Engineering, Southern University of Science and Technology, 518055 Shenzhen, China; 6 College of Architecture and Civil Engineering, Beijing University of Technology, 100022 Beijing, China; 7 The Institute of Hydrogeology and Environmental Geology, Chinese Academy of Geological Sciences, 050061 Shijiazhuang, China; 8 Shaanxi Key Laboratory of Accelerator Mass Spectrometry Technology and Application, 710061 Xian, China; 9 Interdisciplinary Research Center of Earth Science Frontier, Beijing Normal University, 100875 Beijing, China; 10 Open Studio for Oceanic-Continental Climate and Environment Changes, Pilot National Laboratory for Marine Science and Technology (Qingdao), 266200 Qingdao, China

## Abstract

The mid-Pleistocene transition (MPT) is widely recognized as a shift in paleoclimatic periodicity from 41- to 100-kyr cycles, which largely reflects integrated changes in global ice volume, sea level, and ocean temperature from the marine realm. However, much less is known about monsoon-induced terrestrial vegetation change across the MPT. Here, on the basis of a 1.7-million-year δ^13^C record of loess carbonates from the Chinese Loess Plateau, we document a unique MPT reflecting terrestrial vegetation changes from a dominant 23-kyr periodicity before 1.2 Ma to combined 100, 41, and 23-kyr cycles after 0.7 Ma, very different from the conventional MPT characteristics. Model simulations further reveal that the MPT transition likely reflects decreased sensitivity of monsoonal hydroclimate to insolation forcing as the Northern Hemisphere became increasingly glaciated through the MPT. Our proxy-model comparison suggests varied responses of temperature and precipitation to astronomical forcing under different ice/CO_2_ boundary conditions, which greatly improves our understanding of monsoon variability and dynamics from the natural past to the anthropogenic future.

## Abstract (web summary)

The mid-Pleistocene transition is recognized as a shift in paleoclimatic periodicity from 41- to 100-kyr cycles. Here the authors present a unique mid-Pleistocene transition of coupled monsoon-vegetation changes from 23- to 100-kyr cycles, which indicates varied sensitivity of past climate to astronomical and ice/CO_2_ forcing.

## Introduction

The Pleistocene climate is characterized by significant glacial–interglacial changes in high-latitude ice volume^1–3^, global ocean temperature^4^, sea level^5^, and monsoonal climate^6^. All these variables inherently interacted to generate a remarkable transition of the ice-age cycles from 41-kyr to 100-kyr cycles between 1.2 and 0.7 Ma, called the mid-Pleistocene transition (MPT)^2,7,8^. The MPT might be triggered by a nonlinear response to astronomical forcing^9^ induced by a secular CO_2_ decrease and/or progressive regolith erosion^10–12^. This transition is particularly apparent in numerous proxy indicators that are sensitive to changing glacial boundary conditions^3–6,11,13^. At middle and low latitudes, however, the proxies of monsoon-induced wind and hydroclimate changes in the Arabian Sea and East Asia display prominent precession cycles during the middle-to-late Pleistocene^14,15^.

Simulations with different climate models show that global monsoon changes are sensitive to changes in insolation, CO_2_ concentration, and ice volume^16–19^. Synthesis of Chinese loess, paleolake, and speleothem records confirms that the Asian summer monsoon variation was induced by combined effects of astronomical, ice, and CO_2_ forcing^20^. However, the relative roles of the insolation and coupled ice/CO_2_ changes in driving orbital-scale monsoon variability remain controversial due to the spatial divergence and complexity of the proxy sensitivity to precipitation and temperature changes^14,15,19–22^.

Here we present a centennial-resolution δ^13^C record of inorganic carbonate (δ^13^C_IC_) of a thick loess sequence from the northwestern Chinese Loess Plateau (CLP), a region sensitive to orbital-to-millennial-scale monsoon variability^22,23^. Magnetostratigraphy and pedostratigrahpy, together with burial dating, provide a reliable chronology of the loess–paleosol sequences accumulated over the past ~1.7 Ma. Loess δ^13^C_IC_, a sensitive monsoon proxy, offers novel insights into Pleistocene monsoon variability and dynamics. Unlike the conventional expression of the MPT, our results reveal a compelling transition of the dominant rhythm in coupled monsoon–vegetation system from 23-kyr to combined 23-, 41-, and 100-kyr cycles across the MPT.

## Results

### Setting and sampling

CLP climate is characterized by seasonal changes in temperature and precipitation. Summer is the warm and humid season due to the influence of the summer monsoon, which transports heat and water vapor from the low-latitude oceans. The summer precipitation from May to September contributes to the annual precipitation by 60–75% and results in strong pedogenesis of the loess–paleosol sequences. In contrast, winter is the cold and dry season associated with strong winter monsoon wind, which is linked to the Siberian–Mongolian high-pressure system. The winter monsoon transports vast of dust particles from inland Asia to the downwind CLP. Therefore, Chinese loess, as a unique continental archive, can document multi-scale monsoon variability linked to past climate changes in both high- and low-latitude regions^20–26^.

**Fig. 1:**
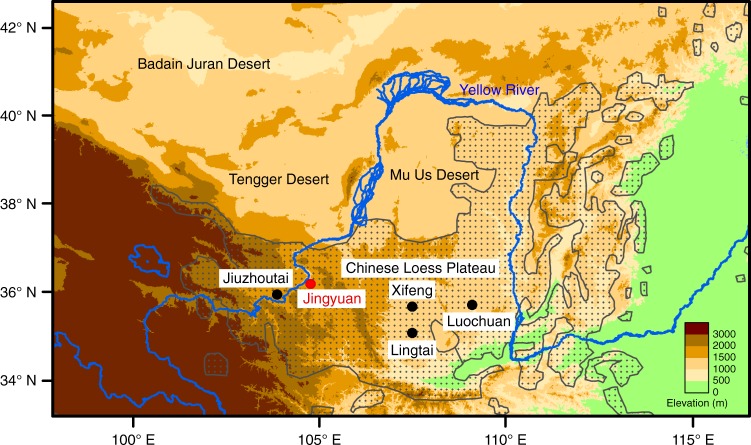
Location of JY loess core and representative loess profiles on the Chinese Loess Plateau (CLP). Black dots denote four classic loess profiles, which have been investigated intensively as the Quaternary stratotype sections^25–31^. Red dot indicates the Jingyuan loess profile accumulated on the ninth terrace of the Yellow River in the northwestern CLP^23,41^

The Jingyuan loess sequence (JY, 36.35°N, 104.6°E, 2,210 m above sea level) is located at the depositional center of modern dust storms on the northwestern CLP (Fig. 1). A 430-m core was retrieved from the highest terrace of the Yellow River near Jingyuan County. The JY core consists of a 427-m loess deposit underlain by a 3-m gravel layer. A 40-m outcrop was excavated nearby to collect parallel samples spanning the last interglacial–glacial cycle. Continuous U-channel and discrete cube samples were taken from the split cores for alternating-field and thermal demagnetization measurements, respectively (Methods). Three quartz samples were chosen from the underlying gravel layer for ^26^Al/^10^Be burial dating (Supplementary Fig. 1). Powder samples were taken at 10-cm intervals for magnetic susceptibility, grain size, and δ^13^C_IC_ analyses.

### Magnetostratigrahy and pedostratigrahy

**Fig. 2:**
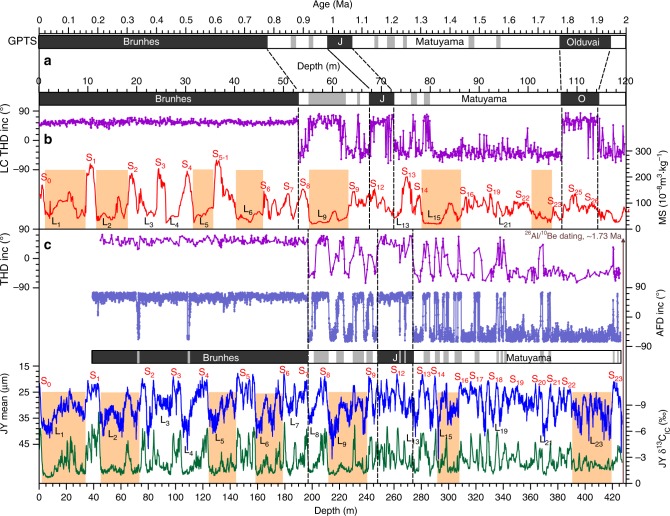
Polarity and proxy variations of the JY loess sequence. **a** Geomagnetic polarity timescale (GPTS)^32,33^. **b** Magnetic susceptibility (MS) and magnetostratigraphy of Luochuan (LC) profile^27,31^. **c** JY δ^13^C_IC_, mean, and magnetostratigrahy. The magnetostratigraphy is derived from inclination results measured by the alternating-field (AFD, light blue) and thermal (THD, purple) demagnetization methods. Dashed lines indicate the positions of paleomagnetic reversals (B/M Brunhes/Matuyama boundary, J Jaramillo, O Olduvai). Orange bars denote thick loess marker layers

Magnetic measurements and demagnetization spectra indicate that the JY loess sediments contain dominantly coarse-grained domain magnetite, which records the primary detrital remanent magnetization (Supplementary Figs. 2 and 3). Paleomagnetic results reveal that the Brunhes/Matuyama (B/M) boundary is recorded at the S_7_/L_8_ boundary and the Jaramillo subchron is located within S_10_–L_13_ (Fig. 2). The magnetostratigraphy of the JY loess sequence is well correlated with that of classic loess profiles from the central CLP^27–31^, e.g., the B/M boundary at the top L_8_ and the Jaramillo subchron between S_10_ and L_13_ (Supplementary Fig. 4). Correlation of the JY magnetostratigrapy with geomagnetic polarity timescale^32^ indicates that the Olduvai subchron is not recorded at the JY section (Fig. 2).

In addition, several normal excursions are evident in the inclination results below the B/M boundary, which either resulted from remagnetization of coarse-grained magnetic particles or are related to genuine geomagnetic excursions^33,34^. Previous studies have deliberately evaluated the fidelity of these relatively short-lived directional anomalies, which could indicate remagnetization caused by physical realignment of magnetic grains in wetter conditions^35^ or by viscous remanent magnetization overprinting on the coarse-grained eolian magnetic particles^36^. The general correspondences between these short-lived directional anomalies and the grain-size curve seems supporting the remagnetization mechanism.

Loess–paleosol alternations can be reliably recognized and correlated throughout the entire CLP using magnetic susceptibility and grain-size variations. While the pedogenesis inferred from the magnetic susceptibility is relatively weak at the JY loess sequence, mean grain-size and δ^13^C_IC_ variations faithfully document the loess–paleosol alternations from S_23_ to S_0_ (Fig. 2), consistent with the pedostratigraphy of other typical Chinese loess profiles^25–31^ (Supplementary Fig. 4). Seven thick loess units (L_1_, L_2_, L_5_, L_6_, L_9_, L_15_, and L_23_) can be readily identified as marker layers for pedostratigrapic correlation. In between these marker layers, JY loess–paleosol alternations from S_23_ to S_0_ can be easily counted from the mean grain-size variations, which are quite similar to large-amplitude grain-size fluctuations of other representative loess profiles (Supplementary Fig. 4).

### ^26^Al/^10^Be burial dating

The concentrations of ^26^Al and ^10^Be in quartz were derived from the measured isotopic ratios (Methods). ^10^Be value was adjusted to match the currently accepted value^37^, and the ^26^Al/^27^Al ratio was normalized to standards^38^. For the Jingyuan location (latitude 36.35°N and elevation 2210 m)^39^, cosmogenic nuclide production rates were estimated to be 150.42 and 22.09 atoms per gram per year for ^26^Al and ^10^Be, respectively. Burial ages were calculated by iteratively solving equations without considering the post-burial cosmogenic nuclide production^40^. Burial ages of three samples from the same gravel layer vary from 1.62 to 1.87 Ma, with uncertainties of 0.14–0.47 Ma (Table 1). The average age of basal gravel layer was estimated to be 1.73 ± 0.13 Ma, which is consistent with the timing of Lanzhou loess sequence on the ninth terrace of the Yellow River^41^. Integrating the paleomagnetic results and the burial dates suggests that the JY loess sequence accumulated after 1.77 Ma.

**Table 1:** Cosmogenic nuclide concentrations and burial age

Sample	^26^Al (10^5^ atoms/g)	^10^Be (10^5^ atoms/g)	^26^Al/^10^Be	Burial age (Ma)
JY-1 (sand)	3.17 ± 0.38	1.02 ± 0.17	3.10 ± 0.63	1.62 ± 0.47
JY-2 (gravel)	4.35 ± 0.24	1.45 ± 0.05	2.99 ± 0.19	1.69 ± 0.14
JY-3 (sand)	2.43 ± 0.41	0.88 ± 0.04	2.76 ± 0.48	1.87 ± 0.40
Average age				1.73 ± 0.13

### Chronology

The loess chronology has been generated using both orbital tuning^25,26^ and a grain-size model^24,42,43^. These approaches result in almost identical ages of the loess/paleosol boundaries from S_8_ to S_0_, matching well with the timing of the glacial/interglacial transitions of benthic δ^18^O stack^3^. During early-to-middle Pleistocene, orbital tuning can provide better constraints on the timing of the loess/paleosol boundaries compared to the grain-size age model approach due to the capacity for more time control points^25,26^. However, orbital tuning can involve obliquity and precession imprints in the age model and thus hamper our assessment of the periodicity changes across the MPT.

**Fig. 3:**
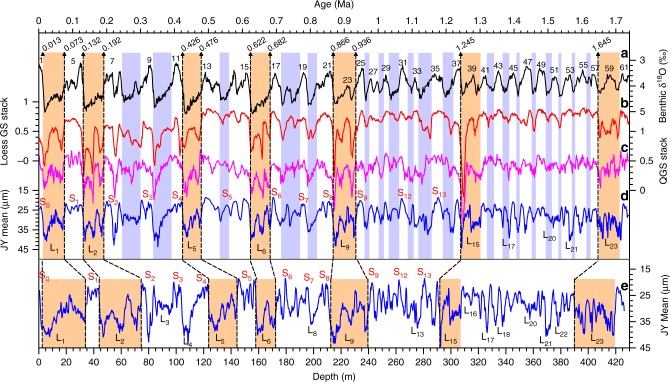
Comparison of JY grain-size time series with stacked loess grain-size and benthic δ^18^O. **a** Benthic δ^18^O stack^3^. **b** Loess grain-size (GS) stack^25^. **c** Quartz grain-size (QGS) stack^26^. **d** JY mean grain-size on age scale. **e** JY mean grain-size on depth scale. Dashed lines denote that the chronology was generated by matching the timing of seven loess marker layers (L_1_, L_2_, L_5_, L_6_, L_9_, L_15_, and L_23_) to stacked grain-size time series. Orange and blue bars indicated the correlation of thick marker layers and other loess units to glacial stages

Owing to the strong similarity of loess–paleosol alternations between the Jingyuan core and other classic loess profiles, a pedostratigraphic age model can be developed using only 12 tie points to match 7 thick loess layers to the stacked grain-size time series^25,26^ (Fig. 3). The age model was refined by interpolation between 12 time controls using the weighted grain-size model^24^. The basal age of the JY loess was estimated to be ~1.73 Ma, and the sedimentation rate varies between 10 and 70 cm/kyr. The age model can be verified by a robust correlation of the Jingyuan grain-size variation with stacked loess grain-size^25,26^ and benthic δ^18^O records^3^ (Fig. 3).

**Fig. 4:**
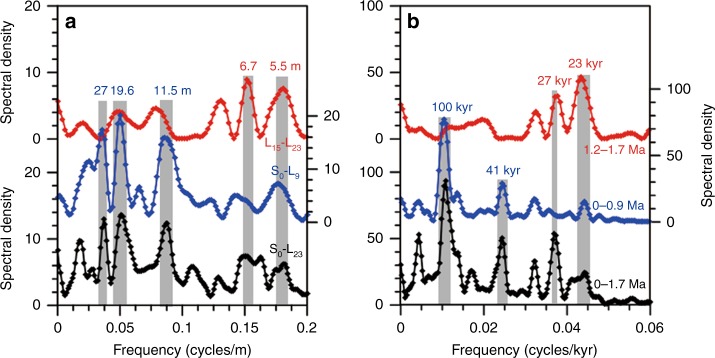
Spectral results of JY δ^13^C_IC_ record. **a** On depth scale, dominant cyclicity changes from 5.5 to 6.7 m/cycle below L_15_ to 19.6-27 m/cycle above L_9_. **b** The dominant cyclicity on age scale is evidently shifted from 23-kyr before 1.2 Ma (below L_15_) to 100-kyr after 0.9 Ma (above L_9_)

Reliability of this age model can be further assessed from the raw depth spectra over three depth intervals (Fig. 4a). The cyclicity ratios (27 m/11.5 m = 2.3 and 11.5 m/5.5 m = 2.1) in the depth spectra are consistent with the orbital ratios (100kyr/41kyr = 2.4 and 41kyr/21kyr = 2.0). Notably, a 5.5-m cycle is evident throughout the entire sequence and becomes dominant below L_15_. Applying a 5.5 m = 21kyr relationship results in a pedostratigraphic correlation of the lower portion to L_15_–S_23_ and a basal age of ~1.73 Ma. Based on a linear depth–time transformation, an intrinsic cyclicity shift is evident from 23-kyr below L_15_ (1.2–1.7 Ma) to 100-kyr above L_9_ (0–0.9 Ma) (Fig. 4b).

Alternatively, applying a 5.5 m = 41kyr relationship could result in a pedostratigraphic correlation of the lower portion to L_15_–S_29_ and a classic MPT from 41- to 100-kyr cycles (Methods and Supplementary Fig. 5). This would require significantly lower sedimentation rate below L_15_ (13.5 cm/kyr) relative to that of the upper portion (25 cm/kyr above L_15_). Such a remarkable change in the sedimentation rate below and above L_15_ is inconsistent with linear age-depth relationships of classic loess profiles on the CLP (Supplementary Fig. 6). Moreover, the basal age (~2.2 Ma) estimated from the alternative pedostratigraphic correlation is significantly older than independent paleomagnetic and burial dating results. Thus we consider that the alternative pedostratigraphic correlation is sedimentologically implausible.

### Loess δ^13^C_IC_ implication

Proxies related to vegetation change such as carbon isotopes of organic matter and inorganic carbonate in Chinese loess have been employed to assess coupled monsoon–vegetation changes^44–47^. Both detrital and pedogenic carbonates are present in the Chinese loess deposits^48,49^. Carbon isotopes of the detrital carbonates from dust source areas over inland Asia vary between a narrow range of −0.9‰ to 0.7‰^22^. Thus the carbon isotope of inorganic carbonate (δ^13^C_IC_) in Chinese loess is controlled mainly by two factors: carbon isotopes of pedogenic carbonate and the proportion of pedogenic carbonates^22,50^. The vegetation in the western CLP is dominated by C3 plants and its carbon isotopes varied by ~2–3‰ due to the biomass changes at glacial–interglacial timescales^44,51,52^. The carbon isotopic difference between inorganic carbonate and organic matter indicates that secondary carbonates roughly contribute two thirds of the glacial–interglacial δ^13^C_IC_ fluctuations (~7‰), which is also attributed to changing vegetation density^22,44^.

To determine the climate-vegetation linkage, the δ^13^C_IC_ results of 20 surface soil samples over the CLP were compared with the precipitation and temperature data over 1981–2010 from the China meteorological data center (Supplementary Fig. 7). The correlation between the δ^13^C_IC_ results and mean annual/summer precipitation is quite similar, whereas its correlation with the mean annual temperature is much higher than the mean summer temperature. Correlations between the δ^13^C_IC_ of surface soil samples and climate variables reveal that while both mean annual temperature and precipitation can affect vegetation growth, mean annual summer precipitation likely plays a dominant role in the vegetation growth over the northwestern CLP. Since temperature and precipitation are strongly coupled over the CLP (i.e., warm–humid in summer and cold–dry in winter), loess δ^13^C_IC_ can serve as a sensitive indicator of the monsoon-induced vegetation change during the Pleistocene.

**Fig. 5:**
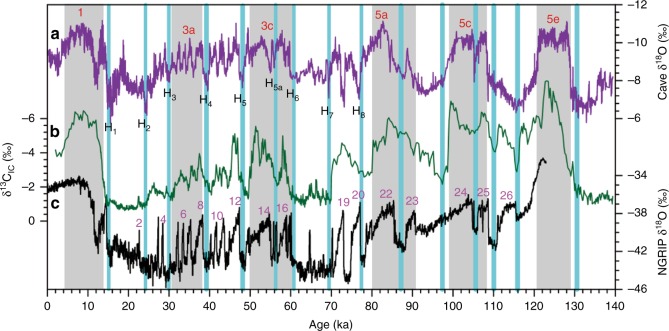
Proxy variations over the last 140 ka from Greenland ice core, Chinese loess, and speleothem. **a** Chinese spleothem δ^18^O record^15^. **b** JY loess δ^13^C_IC_. **c** Greenland ice-core δ^18^O record^53^. Dashed lines denote correlations of positive loess δ^13^C_IC_ and speleothem δ^18^O with low Greenland temperature

The δ^13^C_IC_ records display clear precession and millennial oscillations over the last 140 ka (Fig. 5), identical to those in Chinese speleothem and Greenland ice-core records^15,53^. During marine isotope stage 5 (MIS 5), three negative peaks in the loess δ^13^C_IC_ exhibit a decreasing trend from MIS 5e to MIS 5a, corresponding to changing summer insolation maxima. Our δ^13^C_IC_ records also exhibit stadial–interstadial oscillations. Thirteen Heinrich-like events revealed by the loess ^13^C_IC_ records match well with weak summer monsoon intervals in the speleothem δ^18^O and cold stadials in the Greenland ice core. High similarity between precession-to-millennial variability in Chinese loess, speleothem, and Greenland ice-core records confirms that our loess δ^13^C_IC_ record is sensitive to both high-latitude temperature and low-latitude hydrological changes.

### Sensitivity simulations

To estimate the monsoon sensitivity to various forcings, we use the set of 61 experiments with the climate model HadCM3^54^, which were designed to sample the ensemble of glacial, CO_2_, and astronomical conditions experienced by the land–ocean–atmosphere system during the Pleistocene (Methods). A statistical model (called emulator) is used to interpolate the output of these experiments and to simulate the trajectory of the climate system throughout the Pleistocene. The model results are used to estimate the relative influences of different astronomical parameters (eccentricity, obliquity, and precession)^35^, CO_2_ concentrations (180–280 ppmv)^56,57^, and discrete levels of glaciation ranging from 1 (Holocene-like) to 11 (Last Glacial Maximum)^54^ (Supplementary Fig. 8).

As the loess δ^13^C_IC_ record is affected by changes in mean annual precipitation (MAP) and temperature (MAT), we focused on these two variables averaged over northern China (30–40°N, 104–120°E), where the precipitation change is more sensitive to summer monsoon intensity^19^. The sensitivity analysis was based on the emulator approach^54^. The emulator is evaluated using a leave-one-out cross-validation approach, which consists of predicting the output of one experiment using the emulator calibrated on all the other experiments. Prediction errors for the MAT and MAP over northern China are well calibrated, and about 2/3 of the prediction errors are within one standard deviation (Supplementary Fig. 9).

The calibrated emulator is then used for two purposes. First, we estimated the evolution of these indices throughout the Pleistocene to reconstruct the MAP and MAT curves. The MAP and MAT values are the estimated equilibrium response of HadCM3 for forcing conditions spanning the last 1.7 Ma. The climate evolution estimated following this approach considers thus that MAT and MPT are in quasi-equilibrium with the forcing components, including astronomical solutions^55^, ice levels^3^, and CO_2_ concentrations^56,57^. Next, we estimated the total variances of both MPT and MAT with the emulator, before and after the MPT. The expected variances of these variables caused by the variations of only one or two factors (eccentricity and longitude of perihelion, obliquity, ice, and CO_2_) were estimated by assuming that the other factors are fixed^54^.

### Mid-Pleistocene monsoon transition

**Fig. 6:**
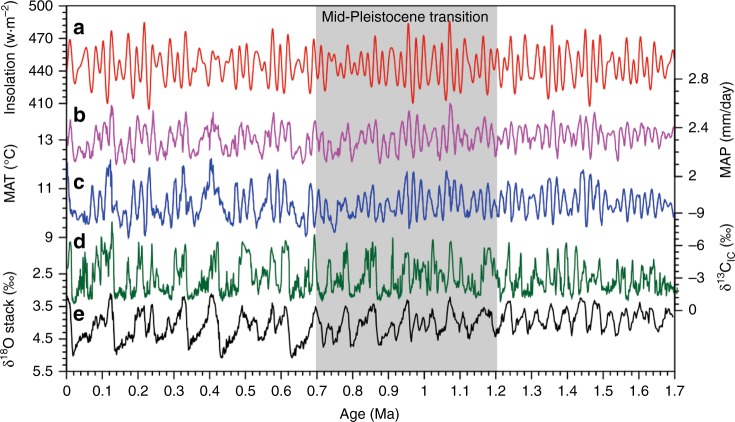
Variations of proxy data and model results over the past 1.7 Ma. **a** Summer insolation at 65°N^55^. **b** Simulated mean annual precipitation (MAP) and **c** mean annual temperature (MAT) over the northern China. **d** JY loess δ^13^C_IC_ record. **e** Benthic δ^18^O stack^3^. The gray bar denotes the mid-Pleistocene transition

The mean grain-size (an indicator of the winter monsoon intensity^25,26^) and δ^13^C_IC_ (a proxy of monsoon-induced vegetation density^22,44^) records display remarkable changes in both frequency and amplitude around two coarsening loess units (L_15_ and L_9_) (Fig. 2). Notably, the δ^13^C_IC_ records show a distinctive transition from rapid oscillations (5.5 and 6.7 m/cycle) below L_15_ to low-frequency fluctuations (11.5, 19.6 and 27 m/cycle) above L_9_, representing an intrinsic shift from 23-kyr before 1.2 Ma to 41- and 100-kyr cycles after 0.9 Ma (Fig. 4). Over longer timescales, the loess δ^13^C_IC_ record exhibits distinctive glacial–interglacial and precessional variability over the past 1.7 Ma, which differs significantly from both summer insolation and benthic δ^18^O (Fig. 6). In the loess δ^13^C_IC_ records, precessional cycles were dominant before 1.2 Ma, followed by a mixing of 23- and 41-kyr cycles during the mid-Pleistocene. After 0.9 Ma, the 100-kyr glacial–interglacial cycles became dominant relative to the already present 41- and 23-kyr periodicities. The durations and rhythms of the interglacial climate varied significantly across the MPT. The past eight interglacials (MIS 21–1) are characterized by reduced precession-band variance and long durations (30–60 kyr) compared to precession-dominated interglacials before the MPT.

**Fig. 7:**
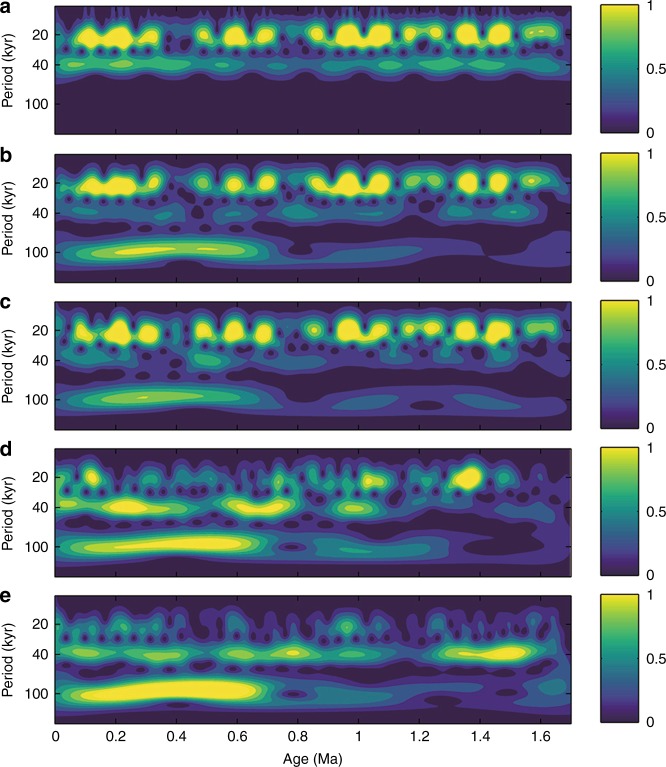
Wavelet power spectra. **a** Summer insolation^55^, **b** simulated MAP, **c** simulated mean annual temperature, **d** loess δ^13^C_IC_, and **e** benthic δ^18^O stack^3^

The timing and duration of the MPT is well illustrated using continuous wavelet transforms (Methods and Fig. 7). Unlike the classical expression of a shift from 41- to 100-kyr cycles revealed by the benthic δ^18^O stack, our loess δ^13^C_IC_ records document a diverse shift of the dominant periodicity from 23- to 100-kyr cycles across the MPT. In the loess δ^13^C_IC_ spectrum, the 23-kyr cycles were dominant before 1.2 Ma and weakened after 0.7 Ma, in contrast to the persistent precession cycles in the summer insolation spectrum. Meanwhile, the 100-kyr power initiated at 1.2 Ma and became dominant after 0.7 Ma. The benthic δ^18^O spectrum, however, shows that the dominant periodicity shifted from 41-kyr cycles before 1.2 Ma to 100-kyr cycles after 0.7 Ma. While the timing and duration of the MPT are similar between terrestrial and marine records, different transitions in the frequency domain point to unique mechanisms governing the East Asian monsoon–vegetation responses to astronomical and coupled ice/CO_2_ forcing.

## Discussion

**Fig. 8:**
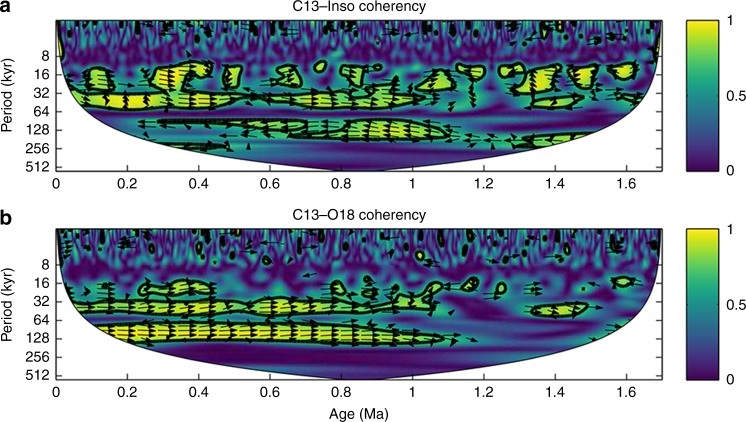
Cross wavelet spectra. **a** JY loess δ^13^C_IC_ vs. summer insolation^55^, **b** JY loess δ^13^C_IC_ vs. benthic δ^18^O stack^3^. Black contours indicate the coherence above 5% significance level

Astronomical parameters and lower boundary configurations are key factors affecting orbital-scale monsoon variability^16–19^. To test the plausible links of the monsoon–vegetation variability to changing insolation and ice sheets, we performed cross-wavelet analyses of the loess δ^13^C_IC_ with summer insolation and the benthic δ^18^O. Cross-wavelet spectra reveal that over the Pleistocene the 23- and 41-kyr cycles in the δ^13^C_IC_ records show strong coherence with the precession and obliquity signals, while remarkable glacial–interglacial fluctuations at both the 100- and 41-kyr periodicities started to be highly coupled with the benthic δ^18^O stack after the MPT (Fig. 8). Different coherency of the loess δ^13^C_IC_ with summer insolation and the benthic δ^18^O mainly in the 100-kyr band suggests that evolving glacial boundary conditions across the MPT may have diminished the climate sensitivity to astronomical forcing by placing strong ice/CO_2_ constraints on the coupled monsoon–vegetation system.

**Fig. 9:**
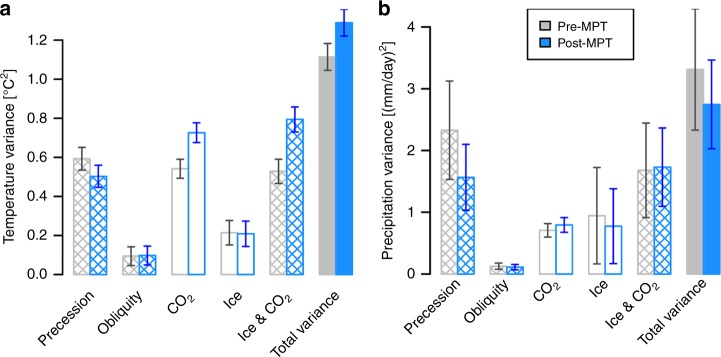
Sensitivity of temperature and precipitation to astronomical, ice, and CO_2_ forcing. **a** Mean annual temperature (MAT) and **b** mean annual precipitation (MAP) responses to precession and obliquity, ice, CO_2_, and coupled ice/CO_2_ forcing. Temperature and precipitation variances are spited into contributions for precession, obliquity, ice, CO_2_, and coupled ice and CO_2_ variation before the MPT (pre-MPT, gray) and after the MPT (post-MPT, blue), respectively. The combined ice/CO_2_ contribution is not estimated by simply adding up the individual impacts of ice and CO_2_ because their effects can partly cancel out each other

The major differences of these forcing factors across the MPT are the ice extent and CO_2_ concentration during glacial maxima^3,58^. Thus sensitivity analyses were based on full glacial–interglacial ranges of the ice levels (1–11) and CO_2_ concentrations (180–280 ppmv) after the MPT and half ranges of the ice levels (1–6) and CO_2_ concentrations (220–280 ppmv) before the MPT. Sensitivity results indicate that the MAT is primarily determined by both precession and CO_2_, and to a lesser extent by ice, and the MAP is mainly driven by precession and less by ice and CO_2_ (Fig. 9). The coupled ice/CO_2_ impact on the MAT is largely attributed to the CO_2_ change, whereas their impact on the MAP is a combination of both ice and CO_2_ changes. Most remarkable differences across the MPT are the decreased sensitivities of both the MAP and MAT to precession forcing and the increased sensitivities of only the MAT to the CO_2_ and coupled ice/CO_2_ forcing. MAT is thus less sensitive to precession forcing after the MPT when the Northern Hemisphere was more glaciated.

Different sensitivities of the MAP and MAT changes to precession, ice, and CO_2_ forcing can lead to diverse manifestations in the amplitude and frequency domains across the MPT (Fig. 6). The simulated MAP over northern China during the last 1.7 Ma shows a persistent 23-kyr periodicity, while the ice/CO_2_ modulation on the MAP became distinctive during glacial periods after 0.9 Ma. By contrast, the simulated MAT change exhibits relatively warm interglacials during 1.5–0.9 Ma and an increase in glacial–interglacial amplitude after the mid-Brunhes event (~0.42 Ma)^59^. Wavelet spectra of the simulated MAP and MAT reveal a distinctive transition from a dominant 23-kyr periodicity before 1.2 Ma to combined 23- and 100-kyr cycles after 0.7 Ma (Fig. 7). Filtered 100- and 23-kyr components in these proxies confirm that the onset of the 100-kyr cycle across the MPT is evident in the loess δ^13^C_IC_, and simulated MAT and MAP, while the 23-kyr cycle is less evident in the benthic δ^18^O than other three proxies (Supplementary Fig. 10). As the MAT and MAP are two important factors affecting the vegetation growth over the CLP, their frequency changes are imprinted in the δ^13^C_IC_ record as a transition from the dominant 23-kyr to combined 23-, 41-, and 100-kyr cycles across the mid-Pleistocene.

In summary, our loess δ^13^C_IC_ time series, for the first time, illustrate a transition of coupled monsoon–vegetation changes from a dominant 23-kyr periodicity to the combined 23-, 41-, and 100-kyr cycles during the mid-Pleistocene, differing from the well-known MPT from 41- to 100-kyr cycles in most marine-based proxy records. While the MPT was usually associated with an increase in ice volume denoted by addition of 41- and 100-kyr variances in the benthic δ^18^O record, sensitivity experiments indicate that the ice impacts on the MAT and MAP over northern China did not vary significantly across the MPT. Rather, the large-amplitude 100-kyr cycle in loess δ^13^C_IC_ is likely attributed to the amplified effect of glacial–interglacial CO_2_ changes on the MAT. Proxy-model comparison reveals that the monsoon–vegetation changes responded dominantly to astronomical forcing before the MPT when the ice and CO_2_ variability was relatively small. After the MPT, however, the precession forcing was attenuated by the coupled ice–CO_2_ effects. Our results highlight varied roles of insolation and coupled ice/CO_2_ factors in driving the temperature and hydroclimate changes from the natural past to the anthropogenic future.

## Methods

### Drilling and sampling

In 2008, we drilled a 430-m core with a recovery rate of 98% at the highest tableland near Jingyuan County. The core consists of a 427-m loess deposits underlain by 3-m gravel layer. Since the recovery rate for the upper 40-m is <85%, we investigated a nearby 40-m outcrop to collect powder samples spanning the last interglacial–glacial cycle. Continuous U-channel samples were taken from the split core sections (40–427 m) for alternating-field magnetization (AFD) measurement. Discrete samples were also collected at 0.5-m intervals using nonmagnetic quartz tubes for thermal demagnetization (THD) measurement. An upcore orientation mark was scribed on the U-channel and tube samples to provide a vertical reference.

### Magnetic measurements

Using a MFK1-FA Kappabridge equipped with a CS-3 high-temperature furnace, temperature-dependent susceptibility (*κ*–*T*) curves were measured in an argon atmosphere from room temperature up to 700 °C with a heating rate of 11 ^o^C/min and then back to room temperature. Hysteresis loops and the first-order reversal curve diagrams were obtained using a MicroMag 3900 automated vibrating sample magnetometer in a maximum field of 1000 mT. Stepwise isothermal remanent magnetization acquisition and direct current demagnetization of saturation isothermal remanence was conducted on the same samples. Hysteresis parameters were calculated after subtracting paramagnetic contributions. A Day plot was used to estimate the domain state and grain size of the magnetic particles.

Stepwise thermal demagnetization was performed on the tube samples from room temperature up to 620–640 °C using a Thermal Demagnetizer (ASC TD-48). The U-channel samples were subjected to progressive AFD at peak fields up to 90 at 2.5–10-mT intervals. The natural remanence of the tube and U-channel samples was measured using a 2 G Enterprises Model 755 cryogenic magnetometer in a magnetically shielded room (<150 nT). Most samples exhibit a weak viscous overprint, which can be removed at the alternating field of 20 mT or at thermal treatment with temperature >300 °C. Principal component analysis, calculated with a least square linear fit, was performed on the demagnetization data. The characteristic remanent magnetization directions were determined by a least-square fitting of the THD results between 300 and 585 °C and of the AFD between 20 and 90 mT, with a maximum angular deviation <15°.

### ^26^Al/^10^Be burial dating

Several kilograms of quartz-bearing sediments were collected from the gravel layer underlying the Jingyuan loess sequence. The quartzose sand and gravel samples were selected for crushing and then the 0.25–0.50-mm fractions were isolated for further purification. Carbonates were dissolved with HCl and magnetic minerals were removed using magnetic separation. The quartz fractions were then leached repeatedly in hot agitated 5% HF/HNO_3_ overnight. The purified quartz fraction was dissolved in 5:1 HF/HNO_3_ and spiked with ~0.3 mg ^9^Be. An aliquot was taken for determining the aluminum content using inductively coupled plasma–atomic emission spectrometry. After evaporation and fuming of the remaining solution in HClO_4_, Al and Be were separated on ion-exchange columns in 0.4 M oxalic acid, precipitated as hydroxides, and transformed to oxides in a furnace at 900 °C. BeO was mixed with niobium and Al_2_O_3_ with copper powder for ^10^Be/^9^Be and ^26^Al/^27^Al measurement by accelerator mass spectrometer at Xi’an AMS Center, Institute of Earth Environment, Chinese Academy of Sciences.

### An alternative age model

A different age model applied to the JY loess–paleosol sequence would generate a shift similar to the classic MPT from 41-kyr to 100-kyr cycles. In this case, the duration of the lower portion (below L_15_) needs be extended from 480 kyr (AGE1, 1.25–1.73 Ma) to 950 kyr (AGE2, 1.25-2.2 Ma) (Supplementary Fig. 5). On the AGE1 model, the lower portion of the JY loess core is correlated to L_15_–S_23_, whereas on the AGE2 model, this portion is correlated to L_15_–S_29_. We prefer the first age model (AGE1) because of two reasons. First, the Olduvai subchron is not recorded in the Jingyuan loess sequence and thus the basal age should be <1.77 Ma. Second, a remarkable shift of the sedimentation rate from 13.5 to 25 cm/kyr around L_15_ based on the JY-AGE2 is significantly different from the relative stable sedimentation rates in several classic loess profiles since the Olduvai subchron (Supplementary Fig. 6). Such a two-fold increase of the sedimentation rate at this single site cannot be explained by either tectonic or climatic factors.

In addition, numerous geomorphologic and paleomagnetic investigations on the terrace sequences of the Yellow River indicate that the loess sequences (S_0_–S_24_) accumulated on the oldest terrace are <1.8 Ma^28,41^ Based on the new terrace classification of the Yellow River^41^, Jingyuan loess sequences (S_0_–S_23_) was accumulated on the ninth terrace, with a rough basal age of 1.7 Ma. While an alternative pedostratigraphic correlation of the lower portion below L_15_ can generate a classical MPT shift from 41-kyr to 100-kyr cycles, the relatively old basal age (~2.2 Ma) and two-fold increase in the sedimentation rates around L_15_ are apparently inconsistent with independent dating results and with age–depth relationships of other classic loess profiles on the CLP, respectively. Thus we think that our preferred AGE1 is more reasonable, as inferred from the paleomagnetic and burial dating results.

### Spectral and wavelet analyses

Spectral analysis was conducted using the Redfit 3.5 software^60^. Wavelet analyses of loess δ^13^C_IC_, simulated MAT and MAT, benthic δ^18^O^3^, and summer insolation^55^ were performed using MATLAB codes^61^ and a Morlet software package^62^. In order to visualize amplitude and frequency modulations, these data sets, except for the summer insolation, were preprocessed to isolate the 100-, 41-, and 21-kyr components using band-passing filters with central frequencies of 0.001, 0.025, and 0.05/kyr and bandwidths of 0.0002, 0.005, and 0.01/kyr before performing the wavelet transforms. The filtered signals are concentrated at three primary astronomical bands and then summed up for wavelet transforms. This preprocessing procedure can reduce the contribution of non-astronomical frequencies in the input signals and therefore improve the calculated power of three astronomical components in the paleoclimatic time series. Wavelet transform coherence was analyzed to assess high-amplitude responses of the loess δ^13^C_IC_, benthic δ^18^O and summer insolation at three orbital bands.

### Sensitivity experiments

We use 61 sensitivity experiments designed to sample efficiently changes in the astronomical configurations, CO_2_ concentrations, and Northern Hemisphere glaciations experienced during the Pleistocene^54^ (Supplementary Fig. 8). Each experiment is 300-year long and we retained averages over the last 100 years for subsequent analysis. The level of Northern Hemisphere glaciations is represented by one variable called “glaciation index,” and which refers to the 1 of the 11 stages of increasing glaciation sampled from the past glacial–interglacial cycle^63^. Level 1 is the Holocene, and level 11 is the Last Glacial Maximum. We note that the configuration choice is such that ice extent grows mainly between stages 1 and 3, while ice thickness growth mainly between levels 3 and 11. Hence, by applying this design to represent ice volume changes during the early Pleistocene, we formulate the hypothesis that area fluctuations of ice sheets before the MPT were similar to after the MPT, but their thickness was smaller.

The sensitivity of the monsoon climate to the full spectrum of climatic conditions experienced during the Pleistocene is estimated using the climate model HadCM3^64^. The atmospheric component dynamics are resolved on a 3.75° × 2.5° longitude–latitude grid, and the oceanic component has a horizontal resolution of 1.25° × 1.25°. The HadCM3 has been shown to capture well enough the monsoon–ENSO interaction^65^ and natural variability of the summer rainfall over China^66^. Compared to observations, the summer subtropical anticyclone in HadCM3 is too strong as in many other models. This circulation anomaly tends to induce much rainfall over the maritime continent, but the error in North China turns out to be very small (<1 mm/day) and thus quite acceptable for our purpose.

In order to generate a forcing-response scenario over the Pleistocene, we scaled the benthic δ^18^O stack^3^ to estimate the glaciation index over the last 1.7 Myr. CO_2_ concentration over the past 0.8 Ma is obtained from Antarctic records^56,57^. The CO_2_ before the 0.8 Ma was extrapolated to follow a linear relationship between ice volume and CO_2_ concentrations over the past eight glacial cycles (Supplementary Fig. 8). The MAP and MAT values are the estimated equilibrium responses of HadCM3 for forcing conditions spanning the last 1.7 Ma by steps of 1 kyr. Validation for the MAT and MAP over northern China suggests that temperature is overall easier to predict than precipitation changes (Supplementary Fig. 9). Indeed, the annual precipitation response results from partly compensating trends in winter and summer. Annual changes are therefore smaller than seasonal changes and more difficult to detect over the internal model variability background. Error bars on the variances of the MAT and MAP correspond to the error variance of the emulator estimates. They combine internal model variability, which affects the values of the 100-year long averages used to calibrate the emulator. Uncertainty might come from the fact that we only have a limited number of experiments to map the sensitivity of HadCM3 to continuous changes in input variables.

## Supplementary information

Supplementary Information

## Data Availability

All relevant data that support the findings of this research are available from the corresponding author on request.
