# Supplementary material for: Diverse manifestations of the mid-Pleistocene climate transition
Source: Nat Commun. 2019 Jan 21;10:352. doi: 10.1038/s41467-018-08257-9 (PMC6341081; doi:10.1038/s41467-018-08257-9)
Supplement: Supplementary file 1 — Supplementary Information [file 41467_2018_8257_MOESM1_ESM.pdf]

Supplementary Information

Diverse manifestations of the Mid-Pleistocene climate transition

Sun et al.

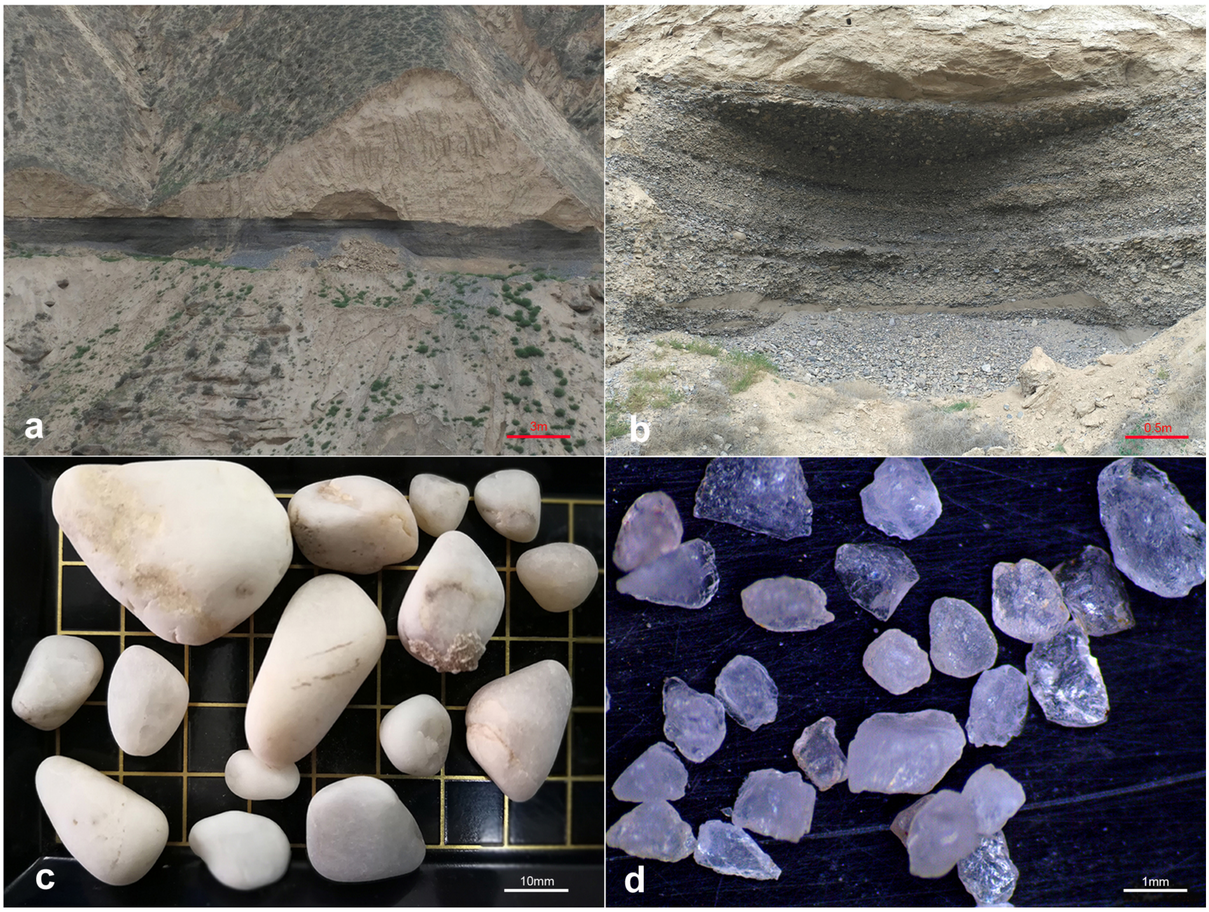

Supplementary Fig.1. Gravel layer below the Jingyuan loess sequence (a,b) and quartz gravels (c,d) for the $^{26}Al/^{10}Be$ burial dating.

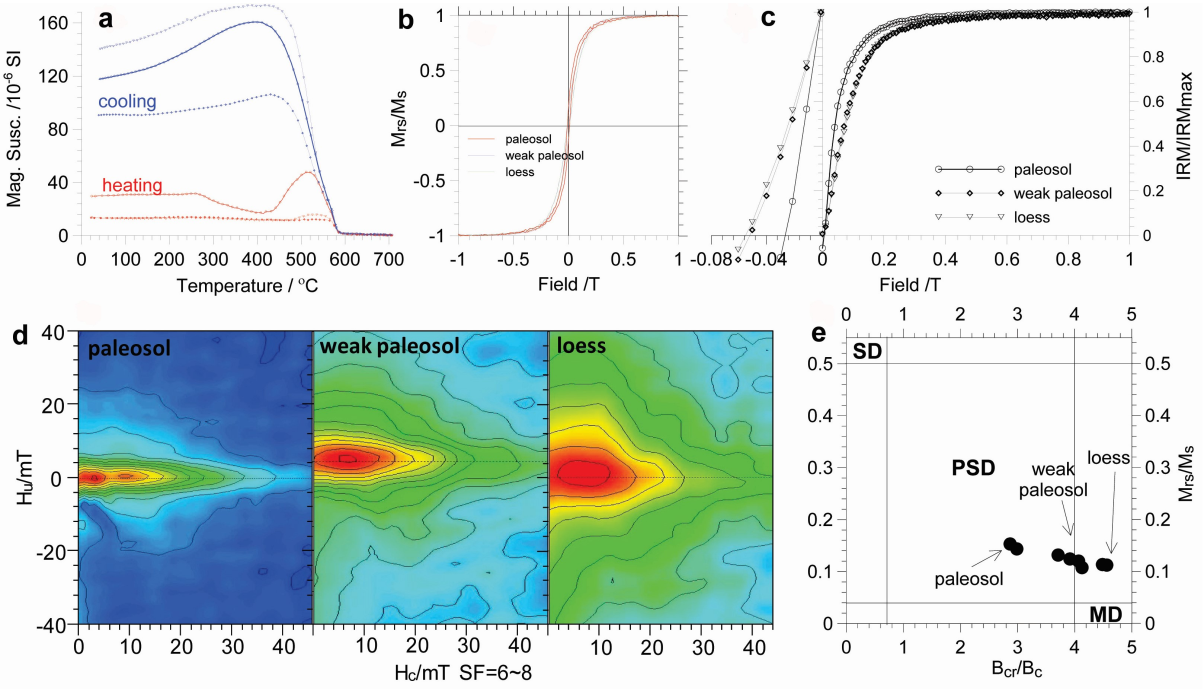

Supplementary Fig.2. Rock magnetism. (a) Temperature-dependent magnetic susceptibility variations of three representative samples. Red and blue lines denote the heating and cooling curves, respectively. (b) Hysteresis loops for three typical samples reveal the presence of both low-coercivity (magnetite and/or maghemite) and high-coercivity minerals (haematite). (c) Stepwise IRM acquisition and demagnetization curves. (d) FORC diagrams of three typical samples exhibit a closed-contour structure in the central part with a peak at about 10-20 mT on the Hc axis and a spreading of the contours along the Hu axis within the range ~20 to 30 mT, confirming the presence of coarse-grained magnetic particles. (e) Day plots reveal that magnetic minerals in both loess and paleosol samples fall within the multidomain (MD) and pseudo-single-domain (PSD) region.

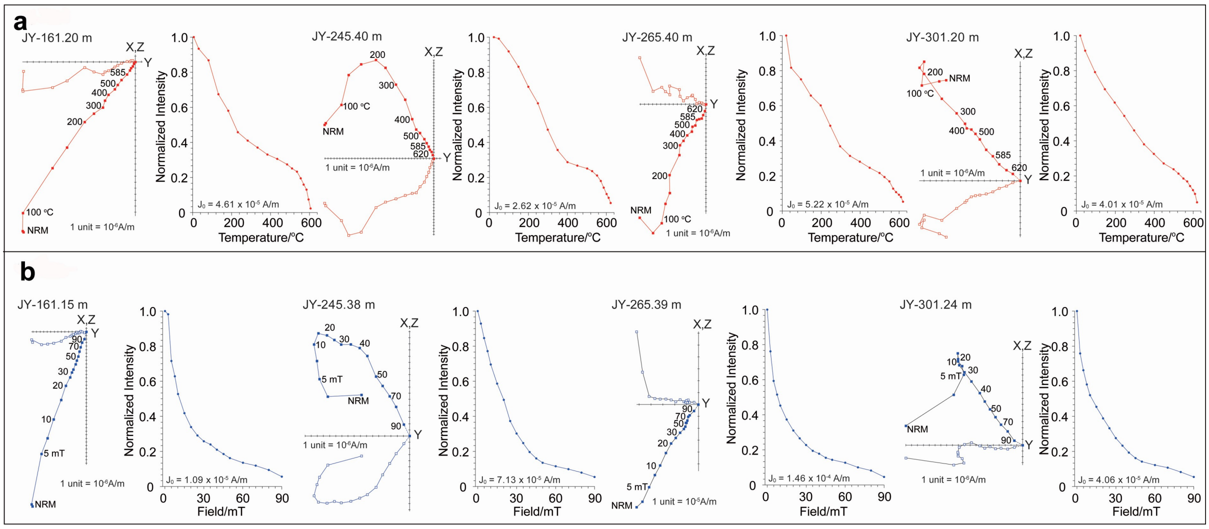

Supplementary Fig.3. Orthogonal projections and normalized intensity decay plots for representative samples. (a) THD results, red solid/open squares indicate the vectors of inclination/declination, the characteristic direction is isolated by about 250-350 °C. (b) AFD results, blue solid/open squares indicate the vectors of inclination/declination, the characteristic direction is isolated by about 20-25 mT. The Z-Y (X-Y) diagrams represent the vertical (horizontal) planes. Since the U-channel and cube samples were taken from the drilling core sections, the azimuth (horizon direction, declination) of the cores cannot be oriented and thus the horizon plane (X-Y coordinate system) is meaningless. However, the dip angle (vertical direction, inclination) of the core can be determined to reflect inclination changes.

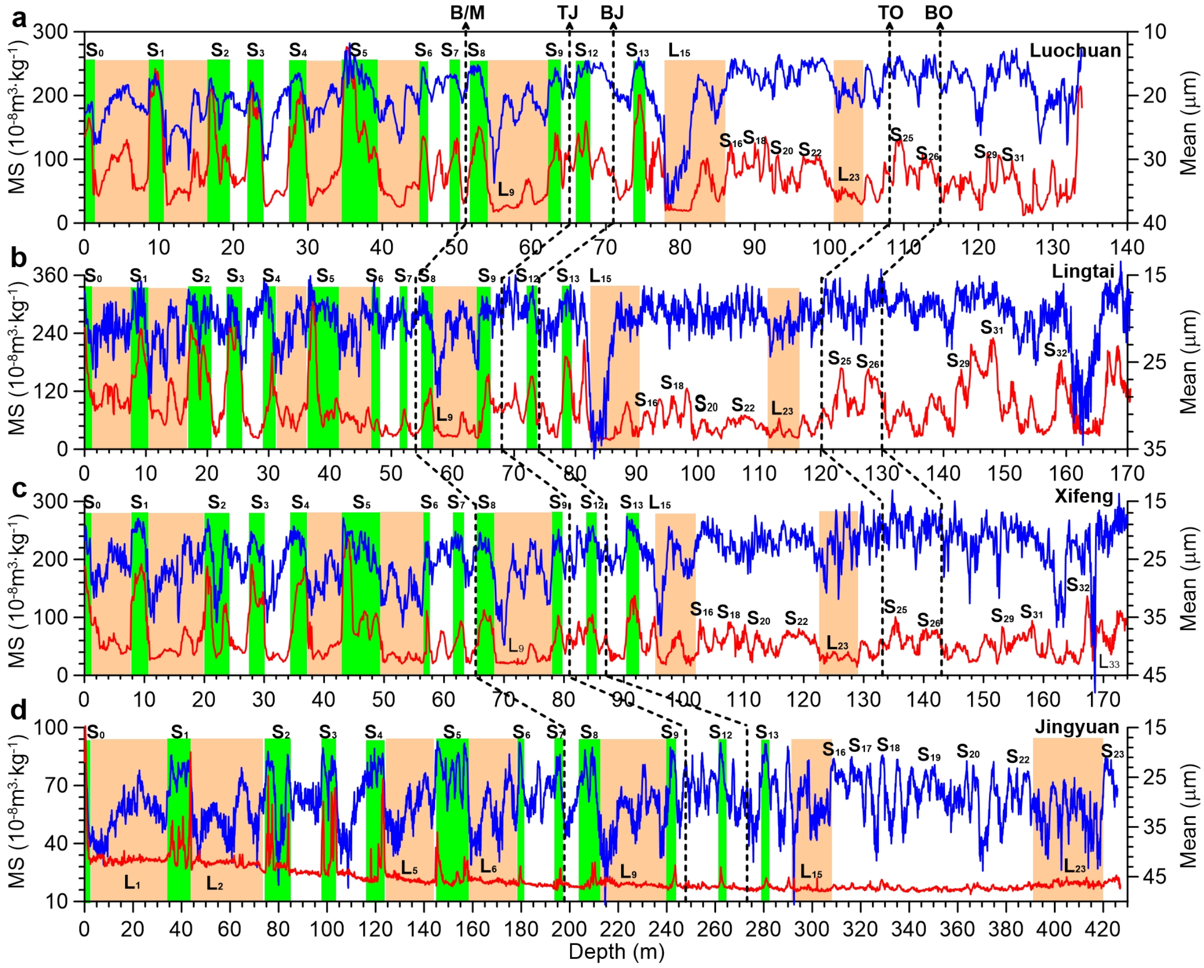

Supplementary Fig.4. Pedostratigraphic and magnetostratigraphic comparison between the Jingyuan loess core and three classic loess profiles from the central CLP. (a) Luochuan[1,2], (b) Lingtai[3], (c) Xifeng[4], and (d) Jingyuan. Dashed lines indicate the positions of paleomagnetic reversals (B/M-Brunhes/Matuyama boundary, TJ-Top Jaramillo, BJ-Bottom Jaramillo, TO-Top Olduvai, BO, Bottom Olduvai). Blue ad orange bars denote pedostratigraphic correlation of paleosols and loess mark layers (L_1, L_2, L_5, L_6, L_9, L_{15} and L_{23}) between the JY and typical loess profiles.

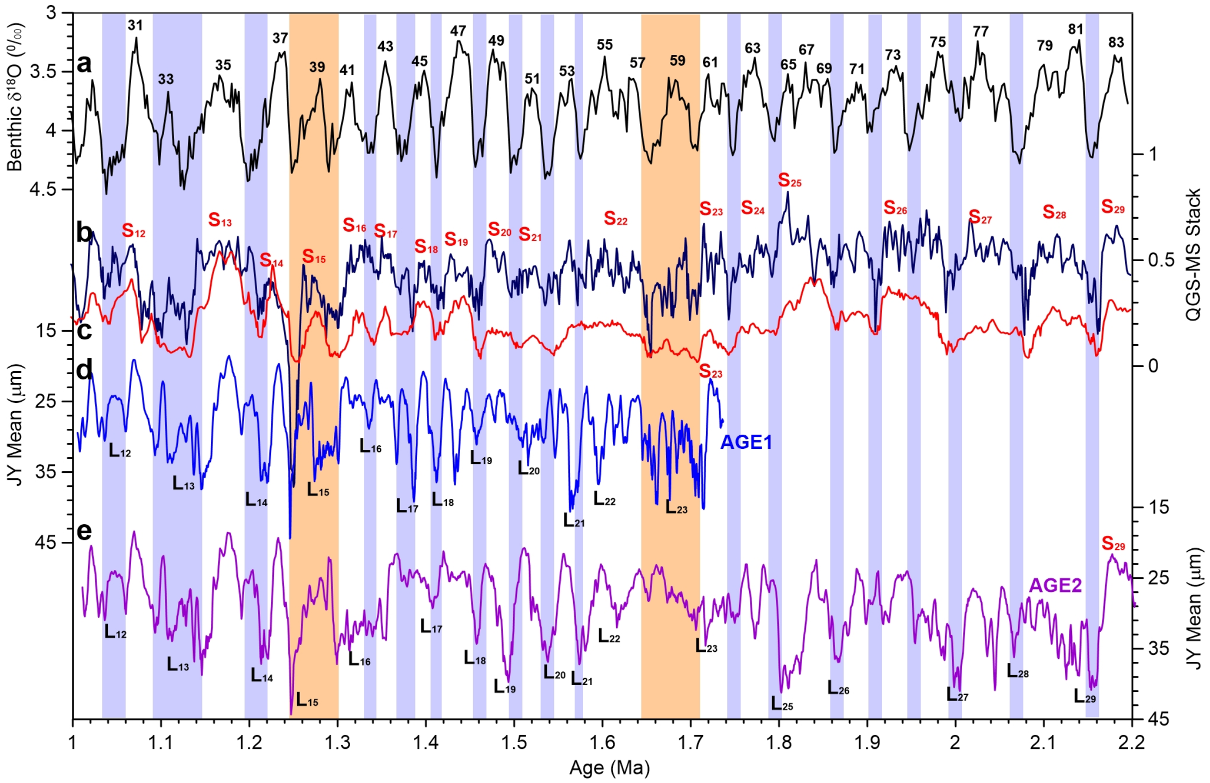

Supplementary Fig.5. Correlations of loess proxies with benthic $\delta^{18}O$ stack. (a) Benthic $\delta^{18}O$ records[5]. (b) Quartz grain-size (QGS) and (c) magnetic susceptibility (MS) stack from two loess profiles from the central Chinese Loess Plateau[6]. (d) JY mean grain-size plotted on AGE1 (L_{15}-S_{23}, 1.25-1.73 Ma). (e) JY mean grain-size plotted on AGE2 (L_{15}-S_{29}, 1.25-2.2 Ma).

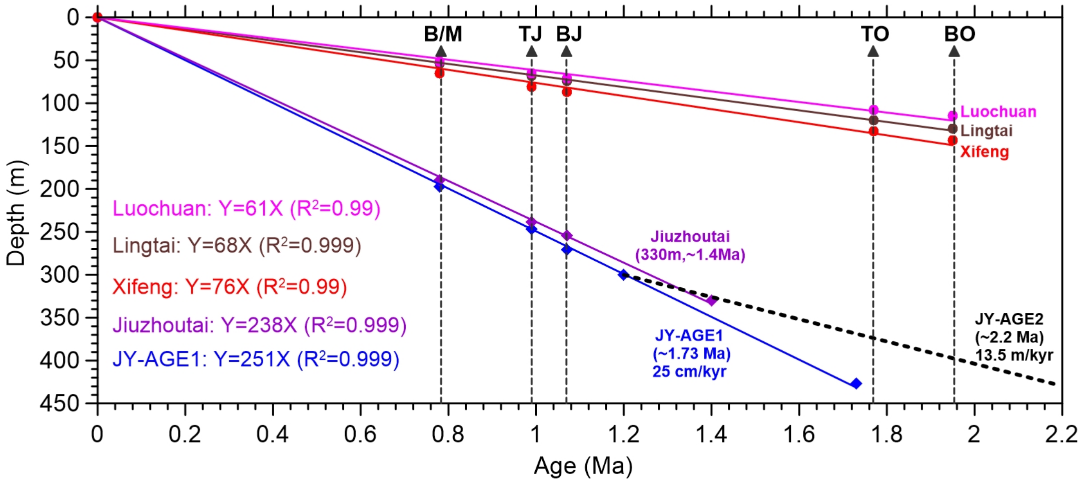

Supplementary Fig.6. Age-depth relationship of five classic loess profiles (Luochuan[1,2], Lingtai[3], Xifeng[4], Jiuzhoutai[7]). The JY core is plotted on two different age models (JY-AGE1, 0-1.73 Ma; JY-AGE2, 0-2.2 Ma).

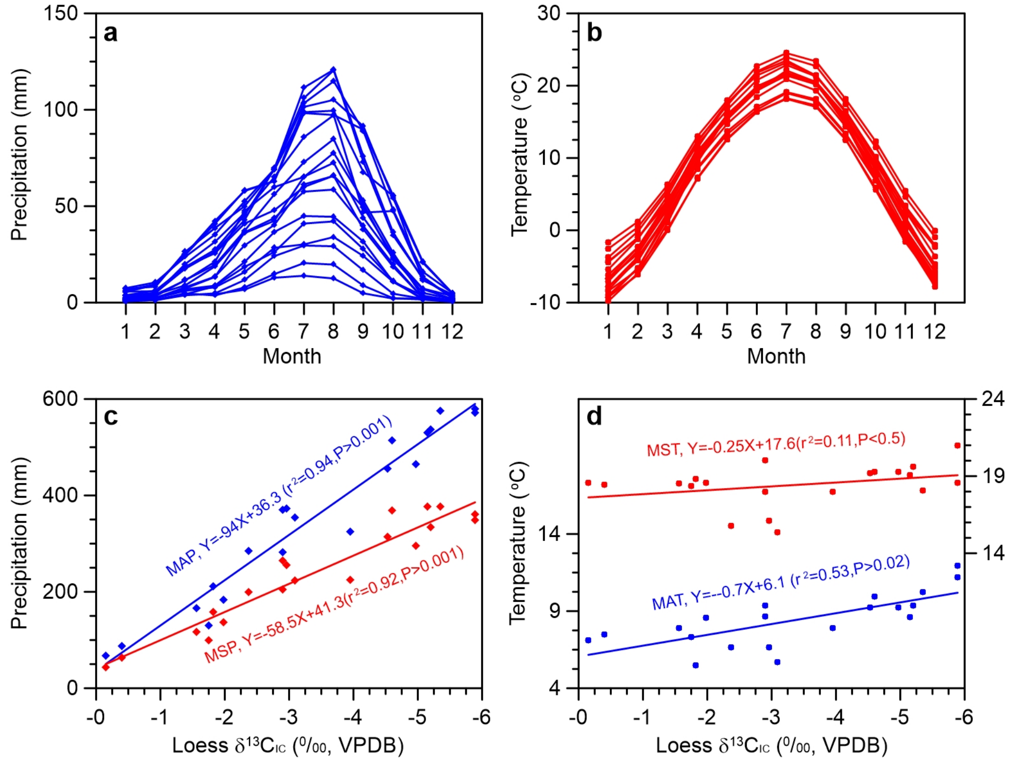

Supplementary Fig.7. Comparison between modern climatic variables and $\delta^{13}C_{IC}$ of surface soil samples over the CLP. (a) Monthly precipitation and (b) temperature changes of twenty sampling sites. (c) Correlation of the $\delta^{13}C_{IC}$ of surface soil samples with changes in mean annual/summer precipitation (MAP/MSP). (d) Correlation of the $\delta^{13}C_{IC}$ of surface soil samples with changes in mean annual/summer temperature (MAT/MST). Monthly averaged precipitation and temperature data over 1981-2010 are from China meteorological data center. Summer season denotes the growing months from May to September.

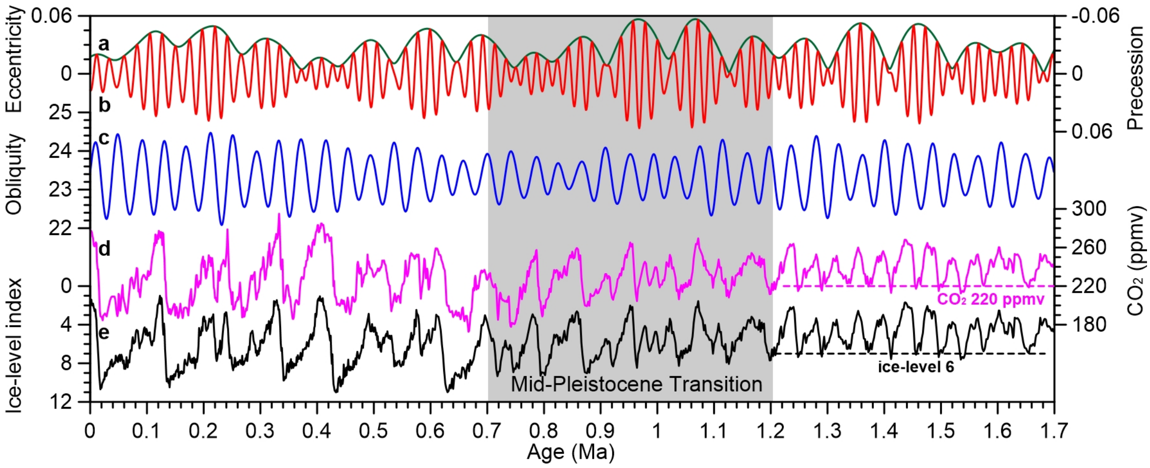

Supplementary Fig.8. Astronomical parameters, ice-level index and CO_2 concentrations in the HadCM3 experiments[8]. (a) Eccentricity, (b) precession, and (c) obliquity from the astronomical calibration[9]. (d) CO_2 concentrations from the Antarctica ice cores[10,11]. (e) Ice-level index[5]. Glacial amplitude of the ice-level (~6) and CO_2 concentration (~220 ppmv) before the MPT were smaller than those after the MPT.

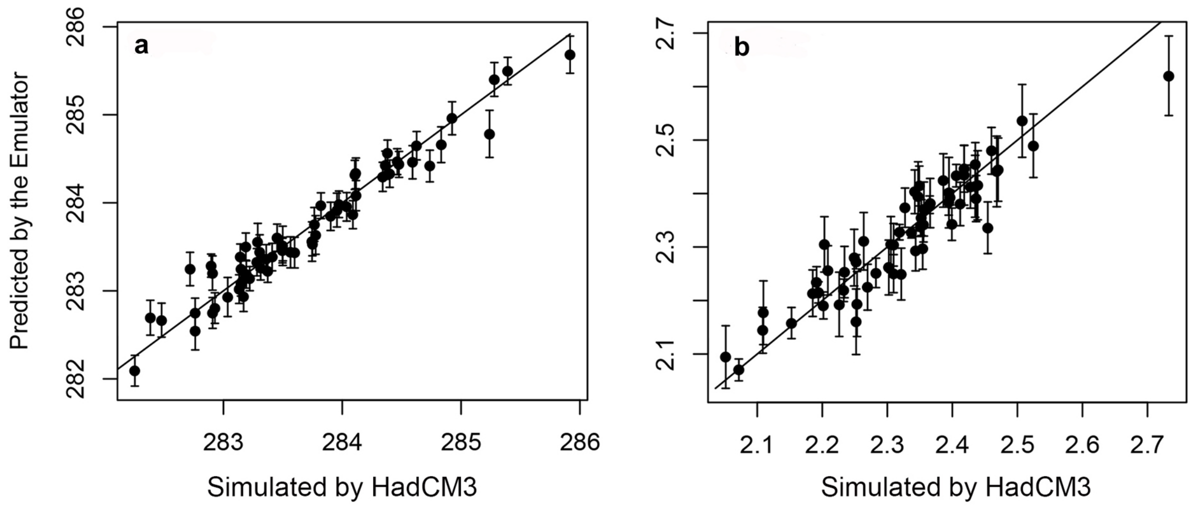

Supplementary Fig.9. Validation of emulator performance for MAT (a) and MAP (b) over northern China. Shown are the values +/- 1 standard deviation predicted by the emulator for each experiment, using the information obtained from the other experiments of the ensemble.

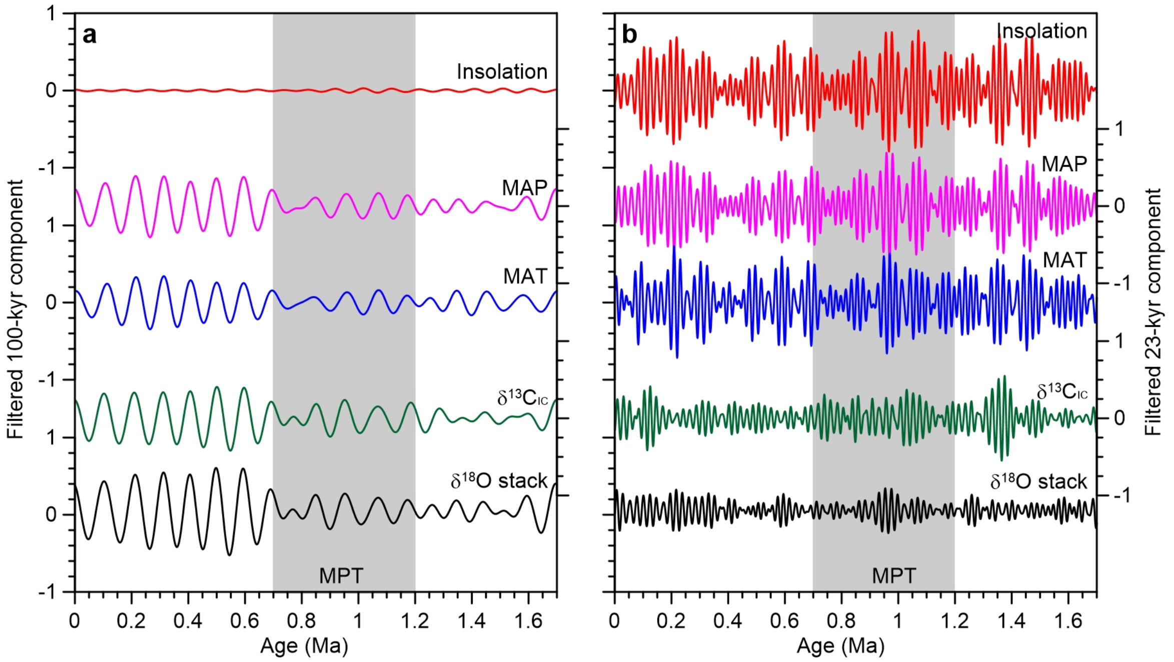

Supplementary Fig.10. Filtered 100-kyr (a) and 23-kyr (b) components of summer insolation[9] simulated MAP and MAT over the northern China, loess $\delta^{13}C_{IC}$, and benthic $\delta^{18}O$ stack[5]. The 100, 41 and 21-kyr components was extracted using band-passing filters with central frequencies of 0.001, 0.025 and 0.05 kyr^{-1}, and bandwidths of 0.0002, 0.005, and 0.01 kyr^{-1}.

Supplementary References.

1. Heller, F. & Liu T. S. Magnetostratigraphical dating of loess deposits in China. *Nature* **300**, 431–433 (1982).
2. Liu W. M., Zhang L. Y. & Sun J. M. High resolution magnetostratigraphy of the Luochuan loess-paleosol sequence in the central Chinese Loess Plateau. Chinese J. Geophis.53, 888–894 (2010).
3. Sun, D. H., Shaw, J., An, Z. S., Chen, M. Y. & Yue, L. P. Magnetostratigraphy paleoclamtic interpretation of a continuous 7.2 Ma late Cenozoic from the Chinese Loess Plateau. *Geophys. Res. Lett.* **25**, 85–88 (1998).
4. Kukla, G., Heller, F., Liu, X. M., Xu, T. C., Liu, T. S., An, Z. S. Pleistocene climates in China dated by magnetic susceptibility. *Geology* **16**, 811–814 (1988).
5. Lisiecki, L. & Raymo, M. A Pliocene-Pleistocene stack of 57 globally distributed benthic $\delta^{18}O$ records. *Paleoceanography* **20**, PA1003 (2005).
6. Sun, Y. B., Clemens, S. C., An, Z. S. & Yu, Z. W. Astronomical timescale and palaeoclimatic implication of stacked 3.6-Myr monsoon records from the Chinese Loess Plateau. *Quat. Sci. Rev.* **25**, 33–48 (2006).
7. Burbank D. W., & Li J., Age and palaeoclimatic significance of the loess of Lanzhou, north China. Nature 316, 429–431 (1985).
8. Araya Melo, P. A.. Crucifix, M. & Bounceur, N. Global sensitivity analysis of the Indian monsoon during the Pleistocene. *Clim. Past* **11**, 45–61 (2015).
9. Berger, A. & Loutre, M. F. Insolation values for the climate of the last 10 million years. *Quat. Sci. Rev.* **10**, 297–317 (1991).
10. Luethi, D., Le Floch, M., Bereiter, B., Blunier, T., Barnola, J. M., Siegenthaler, U., Raynaud, D., Jouzel, J., Fischer, H., Kawamura, K., Stocker, T. F. High-resolution carbon dioxide concentration record 650 00–800 000 years before present. *Nature* **453**, 379–382 (2008).
11. Pertit, J .R., Jouzel, J., Raynaud, D., Barkov, N. I., Barnola, J. M., Basile, I., Bender, M., Chappellaz, J., Davis, M., Delaygue, G., Delmotte, M., Kotlyakov, V. M., Legrand, M., Lipenkov, V. Y., Lorius, C., Pépin, L., Ritz, C., Saltzman, E., Stievenard, M. Climate and atmospheric history of the past 420,000 years from the Vostok ice core, Antarctica. *Nature* **399**, 429–436 (1999).
